# Characterization of *Lophomonas* spp. Infection in a Population of Critical Care Patients

**DOI:** 10.3390/idr16010006

**Published:** 2024-01-26

**Authors:** Francisco das Neves Coelho, João Borralho, Teresa Baptista-Fernandes, Cristina Toscano, Maria Eduarda Carmo

**Affiliations:** 1 Intensive Care Department, Centro Hospitalar de Lisboa Ocidental, Hospital Egas Moniz, 1349-019 Lisboa, Portugal; mecarmo@chlo.min-saude.pt; 2 Infectious Diseases Department, Centro Hospitalar de Lisboa Ocidental, Hospital Egas Moniz, 1349-019 Lisboa, Portugal; jmborralho@chlo.min-saude.pt; 3 Parasitology Department, Centro Hospitalar de Lisboa Ocidental, Hospital Egas Moniz, 1349-019 Lisboa, Portugal; tmfernandes@chlo.min-saude.pt; 4 Microbiology Department, Centro Hospitalar de Lisboa Ocidental, Hospital Egas Moniz, 1349-019 Lisboa, Portugal; mcfigueiredo@chlo.min-saude.pt

**Keywords:** critical care, respiratory failure, parasitology, pneumonia, immunosuppression

## Abstract

*Lophomonas* are flagellated protozoa that have been increasingly associated with upper and lower airway infection in humans. The prevalence and characterization of this disease in the critically ill remains poorly understood. We present a series of eleven ICU patients with confirmed *Lophomonas* spp. identification in respiratory samples.

## 1. Introduction

In recent years, *Lophomonas* spp. has become increasingly associated with upper and lower airway disease in humans [1,2,3,4]. *Lophomonas* is a flagellated protozoa primarily commensal to the gut of insects, like cockroaches and termites, although they may also be found in the environment [5]. The precise mechanism of human exposure to *Lophomonas* spp. remains poorly understood. It has been hypothesized that airway exposure may occur through the inhalation of contaminated dust-containing cysts. Upon excystation within the human host, an acute inflammatory reaction ensues, leading to the development of symptoms [1,2,6]. Many aspects of the interaction between the host and these protozoa remain unknown. It is unclear for how long *Lophomonas* cysts may survive in a dormant state within the human host. It is also uncertain whether the development of lung disease occurs as a consequence of prolonged environmental exposure due to host factors that facilitate excystation, or a combination of both.

*Lophomonas* primarily causes bronchial and pulmonary disease in humans, although cases of upper airway infection, such as sinusitis, have also been reported [7,8]. Clinical presentation, laboratory findings, and radiologic features of *Lophomonas* spp. lower tract infections are nonspecific and cannot be readily differentiated from lung infections caused by common pathogens. Diagnosis may be established by the identification of protozoa in tracheal aspirates or bronchoalveolar samples through direct microscopy in the presence of clinical and radiologic signs of bronchitis or pneumonia [2,4]. A method for diagnostic confirmation through molecular diagnosis has been recently developed but is not widely available [9].

The immunocompromised status of many intensive care unit (ICU) patients may facilitate the acquisition or reactivation of opportunistic infections. Immunosuppressive therapy, invasive procedures, and the deterioration of physiological response due to critical illness are all factors that may facilitate the exposure and development of such disease [10]. Although a strong correlation between low immunologic status and *Lophomonas* infection has been previously established, it has only rarely been described in ICU patients [11,12]. The diagnosis of infection due to uncommon agents in the ICU is usually delayed, likely due to under-recognition, low prevalence, low clinical suspicion, and suboptimal analysis of microbiological samples. This may be particularly true in the case of parasitic infections, which are particularly uncommon in European and North American hospitals [13].

In this report, we present and describe a case series of patients diagnosed with bronchial and lung infections caused by *Lophomonas* spp. during their ICU stay.

## 2. Materials and Methods

We conducted a retrospective analysis of our ICU’s microbiological database from January 2021 to June 2023. We included all patients with a diagnosis of community-acquired, nosocomial, or ventilator-associated pneumonia or tracheobronchitis, where the identification of *Lophomonas* spp. protozoa in the tracheal aspirates or bronchoalveolar lavage was confirmed. This sample included both patients originally admitted to the ICU due to a lower respiratory tract infection and patients who were admitted to the ICU for other reasons but developed new-onset respiratory failure and features of lower respiratory infection during their ICU stay.

A parasitological evaluation of respiratory samples was requested by critical care physicians as part of a bundle to identify opportunistic agents. This request typically occurred in patients exhibiting clinical and radiological features of a lower respiratory tract infection, with no identifiable pathogens in microbiological samples. Occasionally, the microbiologist prompted the request for a parasitological evaluation after identifying mobile elements in a wet mount. 

**Figure A1:**
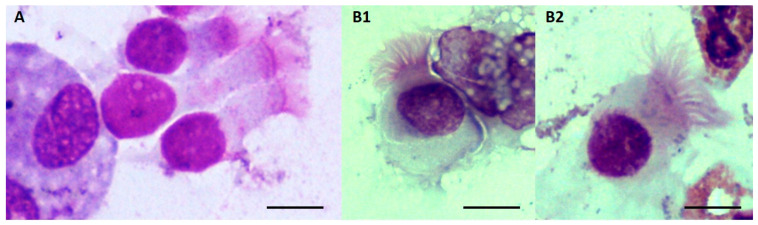
Comparison of morphology between bronchial epithelial ciliated cells (**A**) and *Lophomonas* spp. (**B**). Bronchial epithelial ciliated cells ((**A**), Giemsa stain, amplification ×1000, bar 5 µm) are column-shaped with a nucleus positioned at the basal portion of the cell. Their cilia are short and are positioned above a well-defined terminal band on the apical portion of the cell. *Lophomonas* protozoa ((**B**), Giemsa stain, amplification ×1000, bar 5 µm) may be pyriform (**B1**), ovoid (**B2**), or round in shape. Their flagella originate from a tuft located at the apical end and are long and irregular in length. Cytoplasmic granules and vacuoles are frequently visible, and the nucleus may be located near the apical end.

The protozoa were identified through direct evaluation of fresh microbiological samples. Differentiation between flagellated protozoa and ciliated bronchial epithelial cells was performed by an expert parasitologist, according to known morphological differentiators between the two cell types. These included factors such as cell shape, configuration of cilia/flagella, position of nuclei, and identification of movement on a wet mount [12,14]. An example of the morphological differences between *Lophomonas* spp. and ciliated bronchial epithelial cells is demonstrated in Figure A1 (Appendix A). A demonstration of protozoa movement on a wet mount is also shown on video (see Supplementary Materials). 

We collected data on relevant medical background and comorbidities, the onset of symptoms, clinical features, laboratory and radiographic findings, immunosuppressive therapies, therapeutic approach, and patient outcome. We also specified other agents identified in the same respiratory samples where *Lophomonas* was found. Reported laboratory findings represent the worst value between the onset of symptoms and the definite diagnosis. Reported radiological findings refer to the abnormal findings described in chest tomography and serialized chest radiographies. We expressed the cumulative steroid dose in equivalence to prednisolone to standardize the burden of steroid therapy among patients. “Respiratory insufficiency” was defined as a PaO_2_/FiO_2_ ratio < 300. “Hemodynamic instability” was defined as a mean arterial pressure < 65 mmHg in the presence of cellular dysoxia (represented by serum lactate > 2.0 mmol/L). “Diagnostic delay” was defined as the number of days from the onset of new respiratory symptoms to the identification of *Lophomonas* spp. in microbiological samples. 

A brief statistical analysis, including the average and standard deviation of analyzed variables, was performed using Microsoft Excel 2021^®^.

The present study design was reviewed and authorized by the Ethics Committee of Centro Hospitalar de Lisboa Ocidental (protocol code 2354; date of approval: 19 July 2023).

## 3. Results

Over the course of the 30 months, 1859 patients were admitted to our ICU. *Lophomonas* spp. was identified in the microbiological samples of 11 patients, which corresponds to 0.6% of the patients admitted during that period. Individual patient data can be found in Table A1 and Table A2 (Appendix B and Appendix C). Eight patients were female. The average patient age was 66 years (±13.6 years). Three patients were admitted to the ICU due to community-acquired pneumonia, where *Lophomonas* was the only pathogen identified in microbiologic samples. The remaining eight patients were admitted to the ICU due to other reasons and, within the process of a new-onset pneumonia during their ICU stay, *Lophomonas* was identified in new microbiological samples. Other agents were isolated in the same microbiological sample in four patients, including two viruses (cytomegalovirus, type 3 parainfluenza), two bacteria (*Staphylococcus aureus*; *Klebsiella pneumoniae*), and one mold (*Aspergillus* spp.).

All patients displayed symptoms of acute lung disease. The most common presentation was acute respiratory insufficiency, which was present in ten patients. The average PaO_2_/FiO_2_ ratio for the population was 184 ± 84.4 (minimum 83, maximum 357). An increase in sputum production was observed in nine patients, while fever was present in eight. Three patients developed hemodynamic instability. 

Laboratory findings included leucocytosis (eight patients, average 21,200 × 10^9^/L), neutrophilia (seven patients, average 14,900 × 10^9^/L), and mild to moderate eosinophilia (average 880 × 10^9^/L). C-reactive protein (CRP) was elevated in all patients (average 15.0 ± 13.0 mg/dL), while procalcitonin (PCT) was only significantly increased in one patient. All patients exhibited acute radiological signs on chest tomography, with a wide spectrum of presentations: peribronchial infiltrates (six patients), ground glass opacities (five patients), pleural effusion (four patients), lung consolidation (three patients), and lung abscess (one patient). Although patients exhibited clinical, laboratory, and radiologic findings, there was a significant delay in establishing the diagnosis since the onset of symptoms. The average diagnostic delay was 11 days (±5 days). 

Several patients with *Lophomonas* infection had underlying immunologic impairment. Seven patients had relevant medical history associated with immunodeficiency, including hematologic malignancy (four patients), type 2 diabetes mellitus (two patients), and heart transplantation (one patient). Six patients were receiving high-dose or chronic low-dose steroid therapy, with an average prednisolone-equivalent cumulative dose of 2410 mg (minimum 350 mg, maximum 4670 mg). Another patient was a chronic user of inhaled budesonide for chronic bronchitis. Additional relevant immunosuppressive treatment included active chemotherapy (two patients), everolimus and mycophenolic acid (one patient), and recent bone marrow transplantation (one patient). Only two patients had no identifiable cause for immunosuppression. 

All patients received therapy with metronidazole at varying doses and durations. The prognosis was overall overall, with the resolution of symptoms and radiologic findings in 10 patients. Two patients developed complications from the onset of symptoms until resolution of infection, although it is unclear whether they can be definitely attributed to active *Lophomonas* infection. One patient died due to multiple infectious nosocomial complications and progressive respiratory failure despite adequate treatment.

## 4. Discussion

*Lophomonas* spp. has increasingly been reported and recognized as an emergent pathogen causing upper and lower airway infections. Over the past 15 years, a growing number of case reports and small case series have characterized the clinical and radiological aspects associated with infection caused by this agent [4,12,15]. However, the role and relevance of this protozoa in lower respiratory tract infection and critical illness remains poorly understood.

The clinical findings observed in our patient sample are generally in agreement with the published literature. The clinical features of *Lophomonas* infection or co-infection were nonspecific, with the exception of a higher prevalence of respiratory failure and hemodynamic dysfunction. Common markers of inflammation were frequently observed, although they were nonspecific. We also report a significantly higher number of radiological findings involving bronchi, alveoli, and pleura in chest tomography compared to other reports [1,4,16]. Unfortunately, we did not have a clear record of findings during bronchoscopy, which has also been reported to display abnormalities in the bronchial mucosa [3]. Overall, our patients appear to display a more severe lung infection related to *Lophomonas*, although this could be justified by a higher severity of pre-existing or ICU-acquired illness at the time of the onset of a new respiratory tract infection, such as sequelae from SARS-CoV-2 infection. Nevertheless, our findings demonstrate that *Lophomonas* infection has the potential to generate a significant worsening of respiratory failure in patients already under organ support.

In our patient sample, *Lophomonas* was the only identified cause of lung infection and respiratory failure since hospital admission in three of our patients. This leads us to believe that these protozoa also have the potential to generate an infectious process with significant severity to justify ICU admission per se. It is important to remember that these patients were likely to be under antibiotic treatment at the time of ICU admission, which could have negatively impacted proper microbiological identification. Also, the presumption that a positive therapeutic response to the initiation of metronidazole should be interpreted with caution since metronidazole has antimicrobial activity against other anaerobic or microaerophilic bacteria [17].

Co-infection with both *Lophomonas* spp. and other commonly involved lung pathogens was indeed observed in our patients, but the exact role of protozoa co-infection in pneumonia is unknown. Nearly half of our patients were initially admitted due to acute respiratory failure caused by SARS-CoV-2 pneumonia. While co-infection between SARS-CoV-2 and other viral, bacterial, and molds has been extensively reported [18,19], we are only aware of a single report of a *Lophomonas* spp. and active SARS-CoV-2 infection published by Nakhaei et al. [11]. Comparing our case mix and his case report, we found important similarities, such as a report of respiratory insufficiency and important radiological findings, as well as high-dose steroid therapy as a part of SARS-CoV-2 standard treatment. In addition to the immunosuppression associated with steroid therapy, the authors also suggest that the immune dysfunction caused by SARS-CoV-2 infection may also facilitate the expression of other opportunistic infections [11,20]. Two case reports have been published showing co-infection with *Lophomonas* and cavitated pneumonia secondary to *Mycobacterium tuberculosis* [21,22]. It is likely that the presence of structural damage to normal lung anatomy may also facilitate the propagation of active protozoa and a steady clinical deterioration in these patients through a local inflammatory response, a finding that has been reported already reported by Mokhtarian et al., which reported that the commonest comorbidity on his patient sample was chronic bronchitis [3].

While *Lophomonas* appears to be the cause of significant acute lower respiratory infection in immunocompetent patients [23], it has been mostly reported as an opportunistic infection in patients with impaired immune status. The causes for immunosuppression previously described as associated with *Lophomonas* infection were solid organ and marrow transplantation, long-term steroid treatment, chemotherapy, and HIV infection [24]. Indeed, immunosuppression was highly prevalent among our patient population, which is consistent with previous reports [15]. A significant proportion of patients with *Lophomonas* spp. infection had hematologic malignancy, which, to our knowledge, has not been previously reported. A link between *Lophomonas* spp. and steroid therapy has been consistently reported; however, it is unclear whether steroids facilitate disease progression solely by dampening the host immune response or if they play an active role in protozoa development. It is not clear in the literature whether inhaled steroid therapy may contribute to the development of *Lophomonas* infection, although it has been argued that local impairment of immune response may facilitate the development of opportunistic infection [11,19,21].

We observed a significant delay in establishing the diagnosis of *Lophomonas* infection. However, this delay appears to not have impacted mortality since almost all of our patients fully recovered, despite an average diagnostic delay of 11 days. It can be argued that the favorable prognosis was attributed to organ support provided until a definite diagnosis was obtained but nevertheless, it is plausible that the presence of *Lophomonas* in the lower airway may result in a less aggressive infection with a slower onset of symptoms compared to other agents commonly involved in pneumonia.

Although *Lophomonas* lower airway infections have been primarily reported in patients from China, Iran, Spain, and South America, they may not be limited to specific geographical locations [2,3,4,12,25]. Protozoan airway infections may indeed become increasingly frequent in areas where they have not been previously reported. The lack of awareness regarding the possibility of a protozoa lung infection in specific patient populations in conjunction with the absence of pathognomonic signs of protozoan lung infection and a lack of routine observation of fresh sputum samples contribute to the under-recognition of this agent. We acknowledge that the adequate identification of protozoa carries further diagnostic challenges, although recent publications have clarified the differences between flagellated protozoa and ciliated bronchial epithelial cells [2,6,9]. Molecular diagnostic techniques for the detection of *Lophomonas* spp., however, are likely to be available only in countries with a known high prevalence of this agent.

Persistent clinical, laboratory, and radiologic findings consistent with pneumonia, without documentation of other pathogens in microbiologic samples from the lower airway, and a lack of clinical improvement under empirical antibiotic therapy, should prompt an active pursuit for uncommon pathogens [10]. In our population, appropriate identification was possible through the acquisition of high-quality microbiologic samples and routine direct microscopic evaluation by a specialized parasitologist in suspected cases. 

Adequate collection and processing of microbiological samples plays an important role in the identification of *Lophomonas* spp. Smears stained with Wheatley’s trichrome or Giemsa allow a detailed morphological characterization and differentiation from bronchial ciliated cells. Identification and careful analysis of cell motion in wet mounts is, in our experience, highly valuable in determining the presence of *Lophomonas* in respiratory samples. In order to properly identify the characteristic flagella motions of the protozoa, tracheal aspirates and bronchoalveolar lavages should be immediately sent to the laboratory so that a fresh examination can be performed. 

Improper sample handling, a lack of direct observation of fresh samples, and difficulties in proper differentiation from bronchial ciliated cells may all contribute to the under-recognition and underreporting of *Lophomonas* infection. In our experience, inadequate laboratory management likely plays a role in the underdiagnosis of *Lophomonas* spp. infection. Bacteriological, mycological, and viral tests performed in respiratory samples may be stored at 4 °C, but the characteristic flagellate movements cannot be identified in a sample that has been subject to these conditions. The exact reason for this is unknown, but it is likely that active protozoa cannot survive for long outside of its host or in such adverse circumstances. If screening for parasites is considered necessary by the clinical staff, the laboratory must be informed that the respiratory samples will be collected, so that they will not be refrigerated or left for observation the following morning, which may lead to a false negative.

Molecular diagnosis may improve recognition and diagnosis as it bypasses these limitations associated with sample conservation and protozoa identification, thus increasing diagnostic accuracy [9]. A recent Iranian study retrospectively analyzed the positivity of a *Lophomonas blattarum* molecular screening of 132 frozen bronchoalveolar samples obtained from a number of patients with diverse pulmonary diseases, of which 27% were positive for the presence of *Lophomonas blattarum* protozoa [3]. Positive samples were likely to be from male patients and non-smokers. These findings strengthen the hypothesis that lung infection caused by *Lophomonas* may indeed be more frequent than previously thought. 

While our study provides valuable insights into the clinical characteristics and outcomes of *Lophomonas* infection in critically ill patients, our small sample size restricts our ability to draw definitive conclusions. The inclusion of patients who develop an exacerbation of respiratory symptoms while under treatment for a previously diagnosed acute lower respiratory infection, such as patients with active SARS-CoV-2 pneumonia, induces confusion in terms of determining the degree of respiratory failure and laboratory and radiologic findings, which may actually be attributed to *Lophomonas* infection. The absence of means to establish molecular confirmation limits our ability to confirm *Lophomonas* infection in patients whose microbiological samples did not undergo direct observation under microscopy. Lastly, we report exclusively on adult patients, but the pediatric population appears to be at increased risk of infection as well [4,25].

## 5. Conclusions

Our study highlights the emerging role of *Lophomonas* spp. as a pathogen in patients with acute bronchopulmonary diseases outside of its endemic areas. *Lophomonas* appears to play a role in the development and exacerbation of respiratory failure in patients admitted to the ICU, especially patients with impaired immune status due to underlying comorbidities or immunosuppressive therapy. Patients with *Lophomonas* lung infection within the ICU appear to experience favorable outcomes when appropriate treatment is initiated. However, a lack of awareness results in a delayed diagnosis, and the lack of appropriate screening tools limits an accurate assessment of *Lophomonas*’ true prevalence and clinical impact. These findings highlight the need for increased awareness among ICU physicians regarding parasitological etiologies of pneumonia in hospitalized patients, especially among those with impaired immune function.

## Data Availability

All authorized patient data used in the development of the present research has been included in this manuscript (Appendix B and Appendix C).

## Supplementary Materials

The following supporting information can be downloaded at https://www.mdpi.com/article/10.3390/idr16010006/s1, Video S1: *Lophomonas* spp. displaying circular motion in wet mount. Video S2: *Lophomonas* spp. with flagella displaying wavy motion in wet mount.

## Appendix B

**Table A1:** Clinical characteristics of patients with positive *Lophomonas* sp. identification. Legend: CAP—community-acquired pneumonia; CMV—cytomegalovirus; BMT—bone marrow transplant; T2DM—type 2 diabetes mellitus; SLE—systemic lupus erythematosus; AML—acute myeloid leukemia; * cumulative dose equivalence to prednisolone.

Patient	Age	Sex	Diagnosis at Admission	Diagnosis of *Lophomonas* Infection			Immunologic Status			Clinical Findings			
				Initiation of Symptoms	Other Organisms	Diagnostic Delay (Days)	Steroid Use (in mg) *	Other Immuno-Suppressive Drugs	Relevant Background	Fever	Sputum	Respiratory Insufficiency (Pa/FiO_2_)	Hemodynamic Instability
#1	70	M	SARS-CoV-2 pneumonia	ICU (after SARS-CoV-2 resolution)	No	20	3706	No	No	Yes	Yes	Yes (180)	No
#2	85	M	CAP	Community	No	11	No	No	No	No	Yes	Yes (110)	No
#3	54	M	SARS-CoV-2 pneumonia	ICU (after SARS-CoV-2 resolution)	No	6	1020	No	T2DM	No	Yes	Yes (228)	Yes
#4	65	F	SARS-CoV-2 pneumonia	ICU (after SARS-CoV-2 resolution)	No	8	4670	No	B-cell non-Hodgkin lymphoma	Yes	Yes	Yes (171)	No
#5	62	F	CAP	Ward (hematology)	CMV	14	No	BMT	IgA-lambda myeloma	Yes	Yes	Yes (130)	Yes
#6	80	F	SARS-CoV-2 pneumonia	ICU (after SARS-CoV-2 resolution)	No	5	350	Inhaled budesonide	No	Yes	No	Yes (283)	No
#7	82	F	CAP	Community	No	18	3653	No	SLE, T2DM	No	No	Yes (235)	No
#8	69	F	CAP	Community	No	7	No	Azacitinidine Citarabine Hydroxyurea	AML	Yes	Yes	Yes (129)	Yes
#9	58	F	Lung abscess	Community	*K. pneumoniae* *Aspergillus* sp.	12	2661	Everolimus Mycophenolic acid	Heart transplant	Yes	Yes	No (357)	No
#10	39	F	Fulminant myocarditis	ICU	*Staphylococcus aureus*	10	No	No	No	Yes	Yes	Yes (112)	No
#11	59	F	SARS-CoV-2 pneumonia	ICU (after SARS-CoV-2 resolution)	*Para-influenza* type 3	6	813	Rituximab Cyclophosphamide Doxorubicin Vincristine	B-cell non-Hodgkin lymphoma	Yes	Yes	Yes (83)	No

## Appendix C

**Table A2:** Laboratory and radiologic characteristics, treatment, and outcome of patients with positive *Lophomonas* sp. identification. Legend: CRP—C-reactive protein; PCT—procalcitonin; CT—Computed Tomography.

Patient	Laboratory Findings					Radiologic Findings (Chest CT)					Metronidazole Treatment		Outcome	
	Leuko-cytes (×10^9^/L)	Neutrophils (×10^9^/L)	Eosinophils (×10^9^/L)	CRP (mg/dL)	PCT (ng/mL)	Lung Consolidation	Ground Glass Opacities	Peri-Bronchial Infiltrates	Abscess	Pleural Effusion	Dose (mg)	Duration (Days)	Complications	Survival
#1	13,700	9250	1800	14.5	0.10	Yes	Yes	No	No	No	1000	7	No	Yes
#2	22,100	20,000	1560	18.1	0.60	Yes	Yes	No	No	No	1000	10	No	Yes
#3	23,300	20,600	470	7.93	0.04	No	No	Yes	No	No	500	7	No	Yes
#4	13,000	12,300	30	3.39	0.09	No	No	Yes	No	No	1000	7	Pneumo-mediastinum	Yes
#5	5300	4900	170	29.35	0.70	No	No	Yes	No	Yes	1000	7	No	Yes
#6	19,700	12,600	2400	7.56	0.40	No	No	No	No	No	500	7	No	Yes
#7	9800	8170	60	18.0	1.20	No	Yes	No	No	Yes	1000	10	No	Yes
#8	62,300	19,900	620	8.4	0.46	No	No	Yes	No	Yes	1000	21	No	Yes
#9	38,300	34,400	2030	46.6	10.30	Yes	Yes	No	Yes	No	750	25	Persistent lung infection	No
#10	24,000	20,000	500	6	4.60	No	No	Yes	No	Yes	500	7	No	Yes
#11	1700	1590	60	4.88	0.08	No	Yes	Yes	No	No	500	14	No	Yes
